# Biochar enhances nitrogen use efficiency in lettuce by promoting its metabolic assimilation

**DOI:** 10.1007/s11104-025-07997-0

**Published:** 2025-10-31

**Authors:** Alvaro F. Garcia-Rodriguez, Francisco J. Moreno-Racero, Rosario Álvarez, José M. Colmenero-Flores, Heike Knicker, Miguel A. Rosales

**Affiliations:** 1 Department of Biogeochemistry, Plant and Microbial Ecology, Instituto de Recursos Naturales y Agrobiología de Sevilla (IRNAS-CSIC), 41012 Seville, Spain; 2 Group of Interactions Between Soils, Plants and Microorganisms, Instituto de la Grasa (IG-CSIC), 41013 Seville, Spain; 3 Departamento de Biología Vegetal y Ecología, Facultad de Biología, Universidad de Sevilla, Seville, Spain; 4 Group of Plant Ion and Water Regulation, (IRNAS-CSIC), 41012 Seville, Spain; 5 Department of Stress, Development and Signaling in Plants, Estación Experimental del Zaidín (EEZ-CSIC), 18008 Granada, Spain

**Keywords:** Biochar amendment, *Lactuca sativa* L., Nitrogen assimilation, Physiological traits, Vineyard prunnings

## Abstract

**Background and aims:**

Peat replacement with biochar (BC) offers a sustainable strategy in horticultural substrates but its effects on plant nitrogen (N) metabolism and N use efficiency (NUE) remain unclear. This study tested whether vineyard-pruning-derived BC can boost NUE and metabolic activity in lettuce, providing a pathway toward more productive and sustainable horticulture.

**Methods:**

Plant substrates (BC, peat and vermiculite) were prepared in the following proportions (v:v:v): B0 (0:70:30), B15 (15:55:30) and B30 (30:40:30) for growing lettuce (Lactuca sativa L. var. Batavia) under greenhouse conditions for 31 days. We assessed plant growth and physiological traits, quantified N species and calculated NUE parameters and the activities of key N assimilation enzymes.

**Results:**

B30 plants produced 44.2% more biomass and 23.2% larger leaf area than B0, resulting in lower specific leaf area and greater succulence. BC addition decreased available NO₃⁻ and NH₄⁺ in substrate and roots without causing any plant stress symptoms, as chlorophyll content and PSII efficiency remained stable. B30 increased N uptake flux, N utilization efficiency, partial N balance, and N productivity by 31.8%, 34.8%, 27.8%, and 13.8%, respectively, relative to B0, coinciding with enhanced N-assimilation enzymatic activity. Despite lower total N in roots and shoots, protein accumulation increased, indicating more efficient N conversion into organic compounds.

**Conclusion:**

These findings demonstrate the potential of BC-based substrates (especially 30% BC) to enhance lettuce productivity by improving NUE through the stimulation of N assimilation pathway, offering a promising strategy to optimize N-fertilizer needs to support more sustainable agriculture and soil management practices.

**Supplementary Information:**

The online version contains supplementary material available at 10.1007/s11104-025-07997-0.

## Introduction

More efficient food production systems are needed to guarantee sufficient food supply for a growing global population, while simultaneously reducing the environmental impacts caused by intensive agricultural practices and global warming (van Dijk et al. 2021; Islam 2025). More than half of the world’s population is fed by crops cultivated using synthetic N fertilizers. In 2025, global N consumption is expected to reach 116 million tons according to International Fertilizer Association (Cross et al 2025), and it is projected to increase substantially by 2050, depending on population growth, dietary trends, and fertilizer practices (Zhang et al. 2015). Therefore, there is an increasing global interest in optimizing the use of N resources in agriculture and horticulture.

Plant growth processes are primarily regulated by N, due to its key metabolic role and its influence on net assimilation rate, both of which are directly related to yield improvement (Xia et al. 2022). The main assimilable inorganic N forms in plants are nitrate (NO₃⁻) and ammonium (NH₄⁺). These are taken up by the root system where can be partially assimilated and, subsequently, transported to the leaves to be metabolized or stored in vacuoles (Carillo and Rouphael 2022). In the shoot, the N assimilation pathway begins in the cytosol with the reduction of NO_3_^‒^ to nitrite (NO_2_^‒^) by the enzyme NO_3_^‒^ reductase (NR), followed by the reduction of NO_2_^‒^ to NH₄⁺ by NO_2_^‒^ reductase (NiR) in the chloroplast. Afterwards, NH₄⁺ is then incorporated into carbon (C) skeletons derived from photosynthesis via the coordinated action of the enzymes glutamine synthetase (GS) and glutamate synthase (GOGAT) (Masclaux-Daubresse et al. 2010; Xu et al. 2012). The resulting compounds are subsequently used in the cytosol for the biosynthesis of various amino acids and proteins, involving the enzyme glutamate dehydrogenase (GDH) (Rubio-Wilhelmi et al. 2012; Peinado-Torrubia et al. 2023).

The uptake and assimilation of these N forms largely determine N use efficiency (NUE), which is widely considered as a key physiological trait in crops. NUE is defined as the ratio of N recovered in the harvested product in relation to the total N input from fertilizers or organic amendments (Anas et al. 2020). Low NUE values (< 50%) indicate inefficient N utilization and high N-fertilizer losses, while excessively high NUE values (> 90%) suggests soil N depletion, a phenomenon often referred to as ‘N mining’ (EU Nitrogen Expert Panel 2015).

Previous studies have shown that addition of BC to soilless cultivation systems, can improve plant growth and nutrient utilization (Dispenza et al. 2016; Nocentini et al. 2021; Kunnen et al. 2024). However, wood-based BCs, due to their high C:N ratios, does not add significant N input on their own. Thus, its use as amendment and as external N source is limited, consequently fertilizers or other N-rich amendments (*e.g.* compost, manure, etc.) must still be applied (Bonanomi et al. 2017). On the other hand, recent studies indicated that BC addition to peat-based plant substrates can improve NUE of lettuce plants (García-Rodríguez et al. 2022). In this study, it was evidenced that the substitution of 30% peat with BC in fertilized substrates promoted plant growth relative to control (pure peat-based substrate) conditions. Comparable observations have been also reported for other species (Jia et al. 2021; Nocentini et al. 2021; Xia et al. 2022). The enhanced NUE due to the replacement of peat by BC observed in García-Rodríguez et al. (2022) was likely attributed to a better N acquisition by the plants. Consequently, increased NUE after BC application could help to minimize the over-use of chemical fertilizers in agriculture while optimizing the nutrient resources (Pereira et al. 2017). However, in the experiment by García-Rodríguez et al. (2022), nutrient solution was applied during both germination and plant growth. Bearing in mind that during the early development, seedlings receive most of their nutrients from the seeds, the added nutrients accumulated in or leached from the substrate. It was suggested that due to their high porosity and the presence of certain functional groups, BC can adsorb inorganic N and other nutrients and thus optimize their availability for plants, use of N and the N assimilation pathway (Zhang et al. 2019; Khan et al. 2022). However, the precise mechanisms by which BC amendment affects N assimilation and NUE in crops remain poorly understood.

To address this knowledge gap, the present study aimed to elucidate the physiological and biochemical mechanisms associated with the greater performance of lettuce plants after BC amendment. Accordingly, for the pot experiment, pre-cultivated lettuce seedlings were planted on substrate mixtures composed of water rinsed BC:peat:vermiculite (v:v:v, dry matter) in the following ratios: 0:70:30 (B0), 15:55:30 (B15), and 30:40:30 (B30). No higher BC concentrations were tested, as the former study revealed that contributions of 50% BC affected plant growth negatively (García-Rodríguez et al. 2022).

For a better understanding of how NUE can be affected by BC addition, this work assessed general plant physiological responses (plant growth, development and stress symptoms indicators), by the evaluation of: (i) the distribution of N forms in substrate, root and shoots; (ii) different NUE parameters, including the N uptake flux (J_upt_ N), the efficiency in its use (NUE soil), uptake (NU_P_E) and utilization (NU_T_E), partial N balance (PNB), and N productivity; and (iii) the impact of BC on the activity of key N enzymes involved in N assimilation. The facets of this research intended to approach a holistic understanding of BC mechanisms, contributing to optimize N resources in agriculture.

## Material and methods

### Properties of the substrates

The feedstock used for the BC was provided by Caviro-Enomondo (Faenza, Italy) and derived from green waste of vineyard prunings. This material was pyrolyzed at approximately 500 °C. The main particle size of the final BC was less than 5 cm. To remove potentially toxic compounds and decrease the salt concentration, the BC was rinsed twice with double distilled water prior to the pot experiment. Commercial sphagnum peat and vermiculite substrates were both obtained from Klasmann-Deilmann GmbH (Geeste, Germany). A detailed characterization of the BC and peat substrates, including elemental composition (C and N), C:N ratio, EC, and maximal water holding capacity (WHC), is listed in Table 1 and described in García-Rodríguez et al. (2022). For the pot experiment, peat and BC were mixed with vermiculite in the following BC:peat:vermiculite proportions (%, v:v:v, dry matter): B0 (0:70:30), B15 (15:55:30) and B30 (30:40:30). The pH (H_2_O) of substrate mixtures was measured in suspension with distilled water (1:5) with a Crison pH-meter Basic 20 (Barcelona, España), following the method described by de la Rosa et al. (2014) for carbonized material. The supernatant solution was separated by filtration (Whatman N◦ 2 filter) to measure the EC in the filtered solution with a Crison EC-meter Basic 30 +. To determine the WHC, 40 g of each substrate sample was placed on a Whatman 2 filter in a funnel and saturated with distilled water. For 2 h, the water was allowed to percolate through the filter and the funnel. Then, the weight of the moist samples was measured. The weight difference between dry and moist sample was extrapolated for a duration of the experiment of 12 h according to de la Rosa et al. (2014). The percentage relative to the dry weight (DW) of the substrate sample resulted in the value for the maximum WHC.

**Table 1:** BC and peat physical and chemical characteristics

	Peat	BC
TC (mg g^−1^)	510.0	408
TN_soil_ (mg g^−1^)	19.9	0.5
C:N	25.6	817.8
pH	5.7	10.4
EC (µS cm^−1^)	460	1530
BD (g cm^−3^)	0.4	0.3
WHC (% w:w)	118	164

Total carbon (TC) and nitrogen (TN), C:N ratio, pH, electrical conductivity (EC), bulk density (BD), and water holding capacity (WHC) are expressed on a DW basis for the substrates used (*n* = 3). Data previously published in García-Rodríguez et al. (2022), *Horticulturae* 8, 1214

### Experimental design and treatments

Lettuce (*Lactuca sativa* L. var. Batavia) seeds were sown under greenhouse conditions at 24 ± 2ºC/17 ± 2ºC (day/night) and at 60 ± 10% relative humidity (EL-1-USB data logger, Lascar Electronics Inc., Erie, PA, USA). A photoperiod of 16 h/8 h with a flux density of photosynthetic photons flux density (PPFD) of 300–350 μmol m^−2^ s^−1^ (quantum sensor, LI-6400; Li-COR, Lincoln, NE, USA), and a light intensity of 9000–10000 lx (Digital Lux Meter, LX1010B; Carson Electronics, Valemount, Canada) was applied as described in Franco-Navarro et al. (2021). Before sowing, seeds were vernalized in a cold chamber at 4–7ºC for 3–4 days to synchronize germination. After that, seeds were sown in trays with vermiculite by 1 cm depth and incubated in transparent closed boxes to preserve humidity. Vermiculite was used to ensure water availability for early root development. The trays were watered with 200 ml of distilled water to maintain uniform plant growth. At 15 days after sowing (DAS), the most vigorous seedlings were selected and transplanted to pots (4 cm × 4 cm × 10 cm). For each substrate mixture (B0, B15 and B30), 18 replicates were prepared. Plants were watered from that point with a basal nutrient solution composed of 1.42 mM KNO_3_, 0.625 mM KH_2_PO_4_, 0.053 mM K_2_HPO_4_, 2.29 mM Ca(NO_3_)_2_, 1 mM MgSO_4_, 0.1 mM FeNa-EDTA, 0.1 mM H_3_BO_3_, 0.1 mM MnSO_4_, 29 μM ZnSO_4_, 0.11 μM CoCl_2_, 0.1 μM CuSO_4_, 1 μM Na_2_MoO_4_, and 5 μM KI. The solution was adjusted to pH 5.7 with KOH.

Selected plants with similar development and health status across treatments were kept growing under greenhouse and semi-hydroponic conditions until 31 DAS (Fig. S1). Plants were watered every 2–3 days to maintain substrate moisture at 60% of the maximum WHC. Each tray received 200 mL of nutrient solution, replenished on watering days, to maintain water availability until the end of the experiment. Trays were rotated daily in a clockwise direction to ensure uniform light exposure for all plants.

### Determination of plant biomass and leaf parameters

At the end of the experiment (31 DAS), plants were harvested. Shoot and roots were collected separately with scissors, and roots were gently cleaned of substrate residues. Both fresh biomass (FW) and dry biomass (DW) were determined with a weight balance. Leaves from half of the plants were arranged on a white background and photographed for analysing total leaf area (LA) using ImageJ2 Software with a high precision of 99.95–100% (Rueden et al. 2017). Half of shoots samples and roots were freezed-dried to determine DW. The rest of leaf samples were stored at − 80 °C for subsequent biochemical analysis. Water content (WC) was calculated from the difference between FW and DW as described in Barrs and Weatherley (1962). Specific leaf area (SLA) was calculated as total LA divided by total leaf DW (Marcelis et al. 1998). Leaf succulence (S) was calculated as WC divided by LA (Longstreth and Nobel 1979).

### Leaf SPAD index and quantum yield

The photosynthetic status of the plants was assessed by measuring indirectly the chlorophyll content (SPAD) using a portable SPAD 502 Plus Chlorophyll Meter (Spectrum Technologies, Inc., Plainfield, IL, USA). The efficiency of photosystem II (PSII), commonly named as quantum yield (QY), was determined using a portable fluorometer (FluorPen FP-100; Photon System Instruments, Brno, Czech Republic). Both parameters were taken after 25 DAS on 3–5 fully expanded and photosynthetically active leaves per plant.

### Determination of N species content and NUE parameters

The amount of NO₃⁻, NH₄⁺ and organic N in shoots, roots, and substrates were extracted from 100 mg of dried sample mixed with 10 mL of MilliQ water, shaken in an orbital shaker for 2 h at room temperature, and centrifuged at 3,000 rpm for 5 min. Ion concentrations were measured in the supernatant by a colorimetric plate reader (Omega SPECTROstar, BMG LABTECH GmbH, Germany). Shoot and root NO₃⁻ contents were determined by the salicylic-sulfuric acid method (Cataldo et al. 1975). Similarly, NH₄⁺ was determined colorimetrically from the aqueous extraction following a modified microplate method using a 5% hypochlorite buffer reagent prepared with K_2_HPO_4_ and 30 mL of 5% sodium hypochlorite (NaOCl) (Greweling and Peech 1960). Substrate NO₃⁻ and NH₄⁺ extraction was done according to protocols reported by Dahnke and Johnson (1990) and Mulvaney (1996), and later NO₃⁻ was measured following Bremner (1965) and NH₄⁺ following Greweling and Peech (1960). Organic N was determined by using the Kjeldahl method (Bradstreet 1954).

To evaluate the balance between N inputs and crop production, NUE was used as a physiological indicator, following the framework proposed by the EU Nitrogen Expert Panel (2015). However, given that NUE can be interpreted in various ways and each index has different strengths and limitations, it is recommended to use multiple indicators (Congreves et al. 2021). Therefore, in this study, several NUE-related indices were calculated to obtain a comprehensive and accurate estimation of N efficiency. One of the latter is J_upt_ N of a plant, referring to the rate at which the plant takes up the N from the substrate. Thus, it is a measure of the movement and transport of the N across the root surface and into the shoot of the plant (Sánchez-Rodríguez et al. 2010). A further important parameter is NU_P_E associated to the ability of the plant to uptake the N and incorporate it into the root system (Rasmussen et al. 2015). The NU_T_E is a measure of the plant's ability to convert the uptaken N into aboveground dry biomass (Mălinas et al. 2022). The PNB reflects the plant N content per unit of applied fertilizer N. High values indicate N mining whereas low values indicate excessive N application (Congreves et al. 2021). Finally, the NP is an important measure of the efficiency of the N used by plants. It quantifies the amount of dry biomass produced per unit of uptaken N over a certain period (Berendse and Aerts 2016). A higher NP indicates that the plant is able to produce high amounts of biomass per unit of uptaken N. This is desirable for improving agricultural productivity and reducing environmental pollution from excess N fertilizers (Anas et al. 2020).

Global NUE parameters encompassed: Total N content in the plant (TN_Plant_)^(1)^; Total N accumulation (TNA)^(2)^; N uptake fluxes (J_upt_ N)^(3)^; N use efficiency (NUE_soil_)^(4)^; N-Uptake Efficiency (NU_P_E)^(5)^; N-Utilization Efficiency (NU_T_E)^(6)^; Partial N Balance (PNB)^(7)^; N Productivity (NP)^(8)^, and calculated as follows:

1 $$TN_{Plant}=NO_{3^-}-N+NH_{4^+}-N+Organic\operatorname N$$

According to Ríos et al. (2010)

2 $$\mathrm{TNA}={\mathrm{TN}}_{\mathrm{Plant}}\,{Plant\,Biomass}(\mathrm{DW})$$

According to Sorgonà et al. (2006)

3 $$\mathrm{JuptN}=({\mathrm{NO}}_{3^-}-\mathrm{N}+{\mathrm{NH}}_{4^+}-\mathrm{N})_\mathrm{root}+{({\mathrm{NO}}_{3^-}-\mathrm{N}+{\mathrm{NH}}_{4^+}-\mathrm{N})}_{\mathrm{leaf}}+{({\mathrm{N}}_{\mathrm{reduced}})}_{\mathrm{root}}+{({\mathrm{N}}_{\mathrm{reduced}})}_{\mathrm{leaf}}$$

According to Kruse et al. (2007) and revised by by Sánchez-Rodríguez et al. (2010)

4 $${NUE}_{soil}=\mathrm{Biomass}\,(DW)/\text{Available }N$$

According to Moll et al. (1982) and revised by Congreves et al. (2021)

5 $${NU}_{P}E={\mathrm{TN}}_{\mathrm{Plant}}/\text{Root }(DW)$$

According to Elliott and Läuchli, (1985)

6 $${NU}_{T}E=\text{Biomass }(DW)/{\mathrm{TN}}_{\mathrm{Plant}}$$

According to Siddiqi and Glass, (1981)

7 $$PNB={\mathrm{TN}}_{\mathrm{Plant}}/\text{Fertilizer }N\text{ applied}$$

According to Dobermann, (2007) and revised by Congreves et al. (2021)

8 $$NP=Relative\,Growth\,Rate\,(RGR)/{TN}_{Plant}$$

where $$RGR(\text{g }{\text{g }}^{-1}{\mathrm{day}}^{-1})=(ln {DW}_{final}-ln {DW}_{initial})/{T}_{final}-{T}_{initial}$$

According to Berendse and Aerts, (2016) and revised by Congreves et al. (2021)

### Determination of enzymatic activities and protein content

Frozen leaf tissues (1 g) were ground in a chilled mortar with 2 mL of extraction buffer composed of 21.95 mM Tris (pH 8), 20 µM FAD, 1 mM EDTA-Na_2_, 1 mM DTT, 10 mM Cysteine-HCl, 1 mM PMSF, 5 µm Na_2_MoO_4_·2H_2_O, and 1% (w/v) PVPP and 2 µL of chymostatin as protease inhibitor. The homogenized mixture was centrifuged at 10,000 *g* for 20 min at 4º. The supernatant was used to spectrophotometrically quantify nitrate reductase (NR; EC 1.7.99.4) according to the method of Pinto et al. (2014), nitrite reductase (NiR; EC 1.7.2.1) (Bourne and Miflin 1973), glutamate synthase (GOGAT; EC 1.4.7.1) (Kaiser and Lewis 1984), and glutamate dehydrogenase (GDH; EC 1.4.1.2) (Sánchez-Rodríguez et al. 2011). For glutamine synthetase (GS; EC 6.3.1.2), 0.5 g of frozen leaf tissue homogenized in 1 mL of maleic acid-KOH buffer (pH 6.8), containing 100 mM sucrose, 2 mM DTT, and 20% (v/v) ethylene glycol. The extract was centrifuged at 10,000 *g* for 20 min at 4ºC. The supernatant was used for colorimetric and kinetic GS assays following González et al. (1995). Total soluble proteins were quantified colorimetrically using bovine serum albumin as standard (Bradford 1976).

### Statistical analysis

Statistical analysis was performed using the STATGRAPHICS Centurion XIX software (StatPoint Technologies, Warrenton, VA, United States). The Shapiro–Wilk test served to verify the normality of the datasets. One-way ANOVA was used to determine significant differences between groups. Levels of significance are described by asterisks: *P* ≤ 0.05 (∗), *P* ≤ 0.01 (∗ ∗), and *P* ≤ 0.001 (∗ ∗ ∗). No significant differences are indicated as “ns” (*P* > 0.05). Multiple comparisons of means were determined by the Tukey’s honestly significant difference (HSD) and multiple range test (MRT) tests were included in the afore-mentioned software. Values represent the mean of tested biological replicates for each treatment, and key results were validated across two independent experimental trials.

## Results

### Properties of the substrates

BC addition had no notable impact on the pH but caused a significant rise of EC from 118 to 371 µS cm⁻^1^, corresponding to approximately 68.24% increase when comparing B30 to B0. Whereas the WHC was influenced with 30% BC content up to 4.11% compared to B0 (Table 2).

**Table 2:** pH, electrical conductivity (EC) and water holding capacity (WHC) of the peat:BC:vermiculte mixtures (B0: 0% BC; B15: 15% BC, B30: 30% BC)

	B0	B15	B30	*P-value*
pH	7.8 ± 0.1 a	7.6 ± 0.0 b	7.7 ± 0.0 ab	**
EC (µS cm^−1^)	117.9 ± 8.5 c	214.7 ± 2.7 b	371.3 ± 16.5 a	***
WHC (% w:w)	410.7 ± 2.3 b	426.9 ± 2.0 a	428.3 ± 2.3 a	***

Values are the mean of *n* = 6. Values followed by different letters in columns indicate significant differences according to Tukey’s test. Levels of significance: **P ≤ 0.051; ***P ≤ 0.001

### Plant physiological responses to biochar: growth, morphology, water status, and photosynthetic indicators

**Fig. 1:**
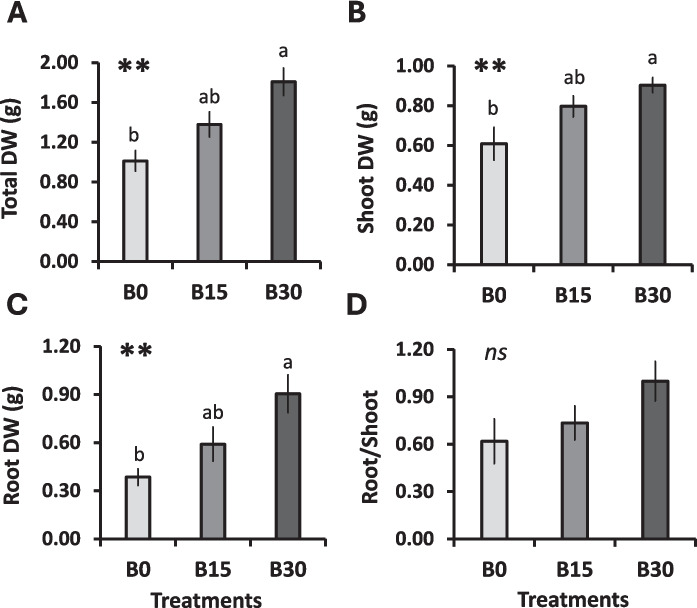
Growth parameters of lettuce plants cultivated with different peat:BC substrates. (B0: 0% BC; B15: 15% BC, B30: 30% BC). (**A**) Total dry biomass (g); (**B**) Shoot dry biomass (g); (**C**) Root dry biomass (g); and (**D**) Root:shoot ratio (%). Values are the mean of *n* = 7–9. Values with different letters are significantly different according to Tukey’s test. Levels of significance: *P* > 0.05 (“ns” not significant differences); **P ≤ 0.01. DW, dry weight

Compared to the plants grown on the substrate without BC (B0), total biomass increased by 26.64% and 44.05% for B15 and B30, respectively (Fig. 1A). Shoot DW rose by 23.63% and 32.62%, and root DW by 34.69% and 57.37%, respectively (Fig. 1B-C). As in our previous study (García-Rodríguez et al. 2022), the greatest shoot and root biomass production was recorded for B30; however, total biomass was higher under the present experimental conditions. This may be associated with the inclusion of seed preincubation or with seasonal changes in greenhouse conditions. Root-to-shoot biomass ratios did not differ significantly among treatments (Fig. 1D), and no signs of physiological stress symptoms were detected, as SPAD values and the quantum yield of photosystem II (QY) remained stable across all conditions (Table 3).

**Table 3:** Water and stress-indicating parameters

	B0	B15	B30	*P-value*
WC (%)	94.95 ± 0.25	95.11 ± 0.32	94.91 ± 0.34	ns
LA (cm^2^)	329.07 ± 23.53 b	385.09 ± 18.56 ab	428.51 ± 16.50 a	**
SLA (cm^2^ g^−1^)	56.17 ± 2.21 a	53.23 ± 1.44 ab	48.21 ± 0.94 b	**
S (mg H_2_O cm^−2^)	34.81 ± 2.11 b	36.73 ± 0.72 b	40.08 ± 1.04 a	*
SPAD index	17.16 ± 0.52	16.54 ± 0.32	17.27 ± 0.39	ns
QY (F’m F’v^−1^)	0.76 ± 0.00	0.75 ± 0.00	0.76 ± 0.00	ns

WC: water content; LA: Leaf area; SLA: specific leaf area; S: Succulence; QY: quantum yield in lettuce plants cultivated under different peat:BC substrates mixtures (B0: 0% BC; B15: 15% BC, B30: 30% BC)

Values are the mean of *n* = 7–9. Values followed by different letters in columns indicate significant differences according to Tukey’s test. Levels of significance: *P* > 0.05 (“ns” not significant differences); *P ≤ 0.05; *P ≤ 0.01

Leaf WC did not exhibit significant differences with respect to increasing BC amendments (Table 3). The LA increased by 14.6% for B15 and 23.2% for B30 compared to B0, whereas SLA decreased and S increased in B30 plants, indicating denser and water-rich foliage, as more water was retained per unit of leaf area (succulence), although total leaf WC remained unchanged (Table 3).

### Effects of biochar on nitrogen content and allocation in plant and substrate

The TN_Plant_ in shoots was only significantly elevated in B15 compared to B30 (Table 4). In contrast, plants grown on B15 and B30 revealed lower TN_Plant_ in roots by 19.43 and 26.36% compared to B0, respectively. For the substrates, BC application significantly reduced TN_soil_, specifically in B30, with a decrease up to 10.86% compared to B0 (Table 4). The TNA content of the plants grown on B15 and B30, raised to 24.08% and 34.11% respectively.

**Table 4:** Total N and N forms in plant organs and substrates

		B0	B15	B30	*P-value*
TN_Plant_ (mg g^−1^ DW)	**Shoot** **Root** **Substrate**	53.80 ± 0.81 ab 28.88 ± 0.35 a 6.63 ± 0.03 a	55.52 ± 0.64 a 23.27 ± 0.66 b 6.17 ± 0.04 b	52.64 ± 0.84 b 21.27 ± 0.38 b 5.91 ± 0.03 c	* *** ***
TN_soil_ (mg g^−1^ DW)					
TNA (mg N)	**Plant**	43.93 ± 3.60 b	57.86 ± 3.10 a	66.67 ± 2.69 a	*
Organic N (mg g^−1^ DW)	**Shoot** **Root** **Substrate**	43.36 ± 1.0 ab 22.32 ± 0.7 a 5.56 ± 0.00 ab	44.53 ± 0.6 a 17.13 ± 1.1 b 5.63 ± 0.00 a	42.27 ± 0.7 b 16.44 ± 0.5 b 5.61 ± 0.00 b	*** *** ***
NO_3_^−^-N (mg g^−1^ DW)	**Shoot** **Root** **Substrate**	10.40 ± 0.82 6.51 ± 0.14 a 1.02 ± 0.52 a	10.95 ± 0.50 5.92 ± 0.26 a 0.52 ± 0.04 b	10.34 ± 0.62 4.77 ± 0.34 b 0.29 ± 0.03 c	ns *** ***
NH_4_^+^-N (µg g^−1^ DW)	**Shoot** **Root** **Substrate**	41.31 ± 4.23 a 57.55 ± 6.02 a 41.84 ± 1.65 a	36.53 ± 3.60 b 34.02 ± 2.02 b 22.16 ± 0.79 b	30.31 ± 2.80 b 30.14 ± 1.78 b 12.48 ± 0.35 c	* *** ***

(TN_Plant_) and soil (TN_soil_) (Total N content; defined as the sum of NO_3_^−^-N, NH_4_^+^-N and Organic N in mg N g^−1^ DW), Total N accumulation (TNA), Organic N, NO_3_^−^-N and NH_4_^+^-N. Values are the mean of *n* = 7–9 ± SE. (B0: 0% BC; B15: 15% BC, B30: 30% BC)

Levels of significance: *P* > 0.05 (“ns” not significant differences); *P ≤ 0.05; **P ≤ 0.01; ***P ≤ 0.001 “homogeneous group” statistics was calculated through ANOVA tests, where mean values with different letters are significantly different according to Tukey’s test

Organic N content in shoots followed a similar pattern to TN_Plant_ (Table 4), showing no significant differences between the plants of the different BC containing mixtures and the control, although B30 exhibited the lowest values among the treatments. In the roots, organic N contents significantly decreased with BC application. A comparable effect was observed for the substrates, where organic N levels increased compared to B0 (Table 4).

The NO₃⁻-N content in the shoots remained unchanged across BC treatments (Table 4). In the roots, a significant decrease was only detected for B30 compared to B0, by 26.73%. In the substrate, NO₃⁻-N levels decreased progressively with increasing BC application. Lastly, as a response to BC application, the NH₄⁺-N contents declined significantly in both shoots and roots reaching the lowest values in B30. The same pattern was observed for the substrates (Table 4).

### Effects of biochar on nitrogen uptake and use efficiency parameters

**Fig. 2:**
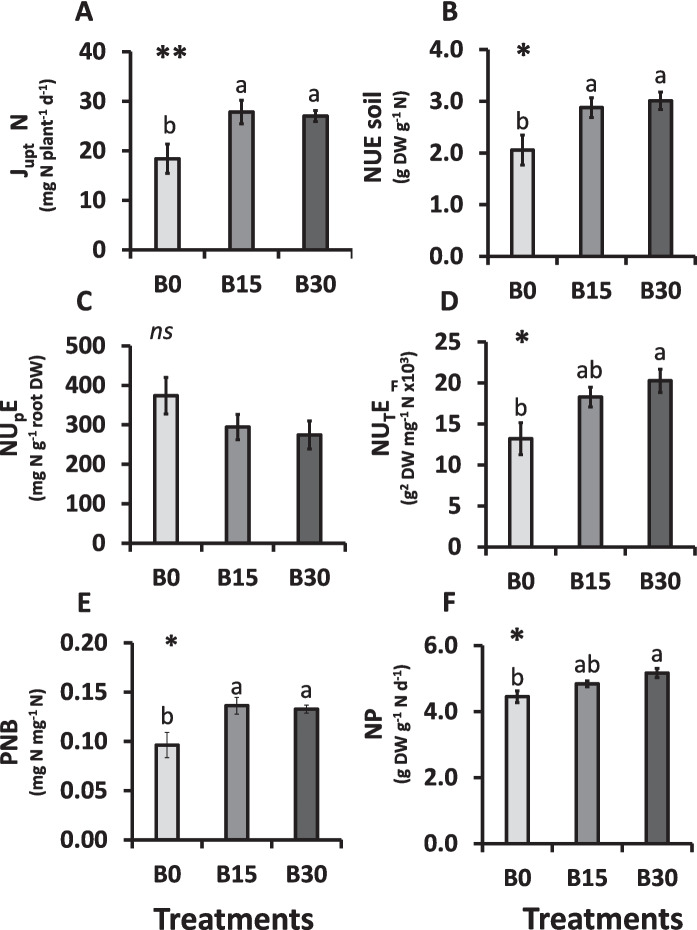
N uptake and NUE parameters in lettuce plants grown under different BC:peat substrate mixtures (B0: 0% BC; B15: 15% BC, B30: 30% BC). (**A**) N uptake fluxes (J_upt_ N); (**B**) N Use Efficiency (NUE); (**C**) N-Uptake Efficiency (NU_P_E); (**D**) N-Utilization Efficiency (NU_T_E); (**E**) Partial N Balance (PNB); (**F**) N Productivity (NP). Mean values ± SE, *n* = 7–9. Levels of significance: *P* > 0.05 (“ns” not significant differences); *P ≤ 0.05; **P ≤ 0.01; “homogeneous group” statistics was calculated through ANOVA tests, where mean values with different letters are significantly different according to Tukey’s test. (Two-column image)

The J_upt_ N increased significantly for the B15 and B30 plants compared to that of the control (B0), indicating that BC addition enhanced N flux capacity of the plants (Fig. 2A). When NUE was evaluated as global index, plants grown on B15 and B30 were more efficient in using N than those of the unamended substrate (Fig. 2B). In contrast, NU_p_E, depicted no significant variation between treatments (Fig. 2C). However, NU_T_E was significantly higher in B30 plants, indicating that this treatment improved transformation of the N taken up by the plant into biomass (Fig. 2D) by 34.8% when compared to B0. The PNB values were also significantly higher in B15 and B30 plants compared to control (Fig. 2E), confirming that the first expressed a greater N retention within the plant system. Finally, NP values increased by 13.8% with BC amendment to B0 plants, suggesting that BC addition improved biomass production per unit of N taken up (Fig. 2F).

### Key enzymatic activities of N assimilation and protein content

**Fig. 3:**
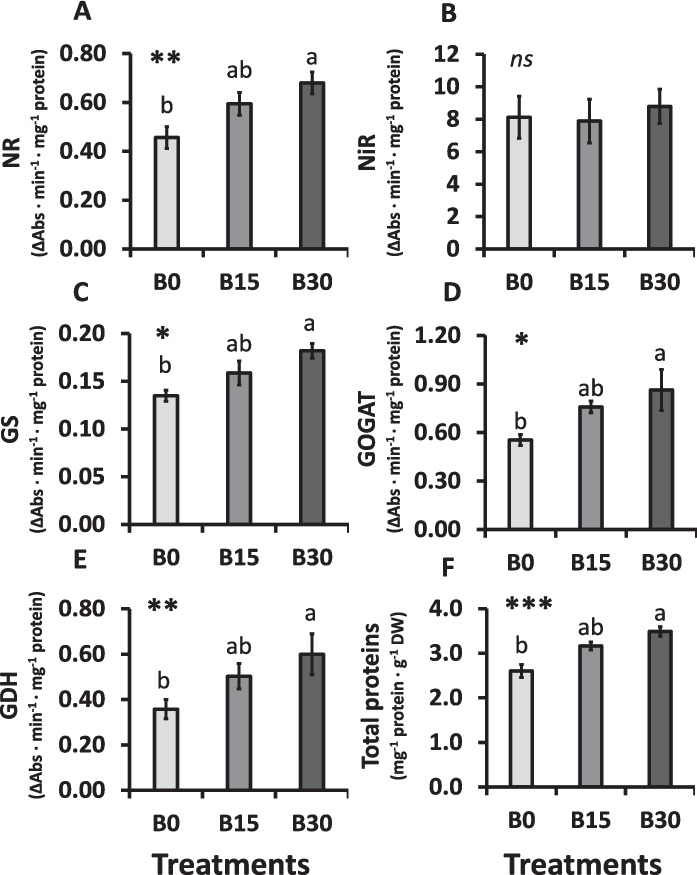
Activity of enzymes involved in the N assimilation in lettuce plants grown under different BC:peat mixtures (B0: 0% BC; B15: 15% BC, B30: 30% BC). (**A**) Nitrate reductase (NR); (**B**) Nitrite reductase (NiR); (**C**) Glutamine synthetase (GS); (**D**) Glutamate synthetase (GOGAT); (**E**) Glutamate dehydrogenase (GDH); (**F**) Total soluble proteins. Mean values ± SE, *n* = 7–9. Levels of significance: *P* > 0.05 (“ns” not significant differences); *P ≤ 0.05; **P ≤ 0.01; ***P ≤ 0.001; “homogeneous group” statistics was calculated through ANOVA tests, where mean values with different letters are significantly different according to Tukey’s test. (Two-column image)

Compared to the plants from B0, those of B30 improved significantly their NR activity, suggesting that BC enhanced the initial step of NO₃⁻ reduction in the assimilation pathway (Fig. 3A). In contrast, NiR activity in the shoots did not show significant differences among BC treatments (Fig. 3B).

The activities of GS, GOGAT, and GDH enhanced progressively with higher BC content in the substrate (Fig. 3C–E). Their upregulation reflects a stimulation of N assimilation downstream of NO₃⁻ reduction. Consistent with this enzymatic response, the content of total soluble protein was significantly higher in B30 plants than in B0 (Fig. 3F), suggesting that the improved assimilation capacity translated into greater incorporation of N into organic nitrogenous compounds such as amino acids and proteins.

## Discussion

### Biochar enhanced lettuce biomass inducing morphophysiological traits

Our study indicates that addition of BC derived from vineyard prunnings to growing media can stimulate lettuce biomass production, as shown by increasing shoot and root growth (Fig. 1A-B). This is in line with previous studies on lettuce and other crop species (Conversa et al. 2015; Fascella et al. 2020; García-Rodríguez et al. 2022; Nurhidayati et al. 2022; Niu et al. 2024).

Aside from affecting the development of the shoots, our results also revealed that BC strongly promoted root growth (Fig. 1C). One possible mechanism is competition for nutrients between BC and plants. Here, the sequestration of nutrients by BC may force root development in search of nutrients after stress signaling has been recognized by plants (Ren et al. 2021; Wan et al. 2023). However, this mechanism seems unlikely to occur in our experiment due to optimal nutrient and water application regimes. In addition, the dry root-to-shoot ratio remaining at 1.00 across treatments supports this assumption (Fig. 1D), suggesting balanced biomass allocation rather than a shift toward root development commonly associated with nutrient availability (Prendergast-Miller et al. 2014). In agreement, no physiological stress symptoms were evidenced: SPAD values (as an indicator of relative chlorophyll content) and the efficiency of PSII (QY) were stable across all treatments (Table 3).

Interestingly, BC also induced distinct changes in leaf morphology, particularly an increase in leaf succulence alongside with a decrease in SLA after addition of 30% BC (Table 3). Similar effects have been reported by Olmo et al. (2014) and Wan et al. (2023) and are commonly associated with thicker and denser leaf structures allowing a more efficient water retention concomitantly with a reduction of water loss through transpiration. Paneque et al. (2016) suggested that reduced stomatal conductance in BC-treated sunflower plants led to improved water use efficiency, which could explain such physiological responses. Moreover, Haider et al. (2015) showed that BC had stimulating effect on soil–plant water relations and photosynthesis. Our results align with those of Franco-Navarro et al. (2021), who reported that greater leaf S in tobacco plants correlated with improved water status, reduced transpiration, and increased biomass under varying nutrient and water regimes. These morphological adaptations, such as reduced SLA and increased S, may therefore contribute to a more favourable physiological state of lettuce grown on BC-containing substrates, potentially supporting higher productivity. Although the underlying mechanisms remain to be fully understood, future research under water-limited conditions are needed to determine whether these responses provide a functional advantage for crop performance in arid environments.

### Nitrogen distribution was shaped by plant uptake rather than biochar retention

In wood-derived BCs, the poor N content prevent their direct use as slow-release N fertilizer. However, the chemical and physical transformation occurring during their production commonly increases the porosity offering additional surface for adsorption or electrostatically attraction of inorganic and small organic N species (Novak et al. 2009; Woolf et al. 2010). It has been shown that such BC is able to capture and store plant-available N (Gao et al. 2019) preventing its leakage or loss through volatilization. Comparably, it has been reported by Castejón-del Pino et al. (2023) that the combination of BC and urea, as external N source, can act as a slow-release N fertilizer since the urea can be temporarily immobilized within the BC´s pores. Indeed, the majority of studies on the implication of BC into N fertilization are now focused on the effects of co-applied N fertilizers and BCs, as BC-based fertilizer. However, in this context, one has to bear in mind that the N-retention efficiency of the BC is in competition with the ability of the plant to uptake nutrients leading to a N deficiency for plants.

In this work, preferential storage of inorganic N-forms (i.e. NO₃⁻ or NH₄⁺) within the BC was not evidenced (Table 4). In addition, the continuous provision of N fertilizer solution to the plant substrates under semi-hydroponics conditions was equal for all treatments and allowed enough nutrient supply. Thus, other mechanism than preferential N storage must be responsible for the improvement of plant performance after BC addition. In the contrary, both inorganic N species decreased in BC-amended substrates and roots, and NO₃⁻-N levels remained unchanged in the shoots. This is in contrast with our previous study, where direct sowing on BC containing substrates and early nutrient supply led to NO₃⁻ accumulation in the substrate (García-Rodríguez et al. 2022). This is best explained by the fact that during the early growing phase, seedlings relied primarily on N reserves provided by the seeds, as observed by Finch-Savage and Bassel (2016). Consequently, the N fertilizer provided via irrigation during this stage could not be taken up immediately by the plant but was adsorbed and retained by the BC through sorption and sequestration processes (Fidel et al. 2018; Ibrahim et al. 2020).

In contrast, in the present study, BC exposure and N fertilizer application began only after seedling establishment, as plants were transplanted into BC-amended substrates following germination. Although both experiments received the same nutrient supply after this stage, less N was retained in the BC under the current conditions, while the young plants demanded similar amounts of inorganic N. Nevertheless, both experiments suggest that BC enhances N uptake and assimilation in plants, although the mechanisms behind this remain an open research question.

### Biochar improves nitrogen use efficiency by enhancing nitrate utilization

As explained before, NUE can be sub-divided in several indicators that together provide a global understanding of the N acquisition and internal utilization. The indicator J_upt_ N (Fig. 2A) increased in the plants grown on BC substrates, reflecting a higher accumulation of N in plant tissues. However, this may imply a greater efficiency of N acquisition of the roots from the soil, but could also be caused by a greater biomass production in BC amended substrates, since NU_P_E did not differ significantly among treatments (Fig. 2C).

The overall NUE soil index (Fig. 2B), which integrates both acquisition and utilization, was enhanced by BC additions, especially at a rate of 30%, suggesting that plant biomass production per unit of supplied N was augmented. This aligns with the observation that lettuce plants from the B30 treatment achieved significantly higher NU_T_E compared to B0 treatment (Fig. 2D), indicating that the used BC can improve transformation of absorbed N into plant biomass once allocated in the shoot. Yuan et al. (2018) reported that the addition of BC and the amount of N absorbed and used by crops are not always necessarily correlated, hence the relevance of assessing both NU_P_E and NU_T_E parameters. The lack of significant differences in NU_P_E among treatments, whereas they achieved significantly greater NU_T_E (Fig. 2D), evidence the idea that the addition of BC to the substrates improved biomass production by positively affecting NO_3_^−^-N utilization once it is allocated into the shoot.

Further insights are provided by the PNB indicator (Fig. 2E), which describes the amount of N retained in the plant per unit of applied fertilizer. This value was significantly higher in plants from BC containing substrates compared to that from the control, revealing a more efficient use of external N inputs by the first. Importantly, shoot NO₃⁻-N contents remained unaffected by the treatments, indicating that BC improved internal N economy without increasing NO₃⁻ accumulation in edible tissues. This is an important consideration since NO₃⁻ can have toxic impacts for humans if consumed excessively (Colla et al. 2018). A further import information can be extracted from the variation of NP with BC addition (Fig. 2F). Higher BC contents in the substrate can be related to a higher plant biomass production per assimilated N. This observation certainly can be of interest for farmers aiming in improving agricultural productivity without excess use of N fertilizers (Anas et al. 2020).

### Biochar enhances enzymatic nitrogen assimilation

The analysis of NUE parameters indicated that addition of BC has an impact on the metabolic pathways involved in N assimilation in lettuce plants. In recent years, several studies have begun to investigate the effect of BC on key N-related enzymes like NR, NiR, GS, and GOGAT (Cao et al. 2019; Saffeullah et al. 2021). However, only few of these studies have directly linked these enzymatic activities with NUE improvements (Chattha et al. 2022; Khan et al. 2022; Zhao et al. 2023).

**Fig. 4:**
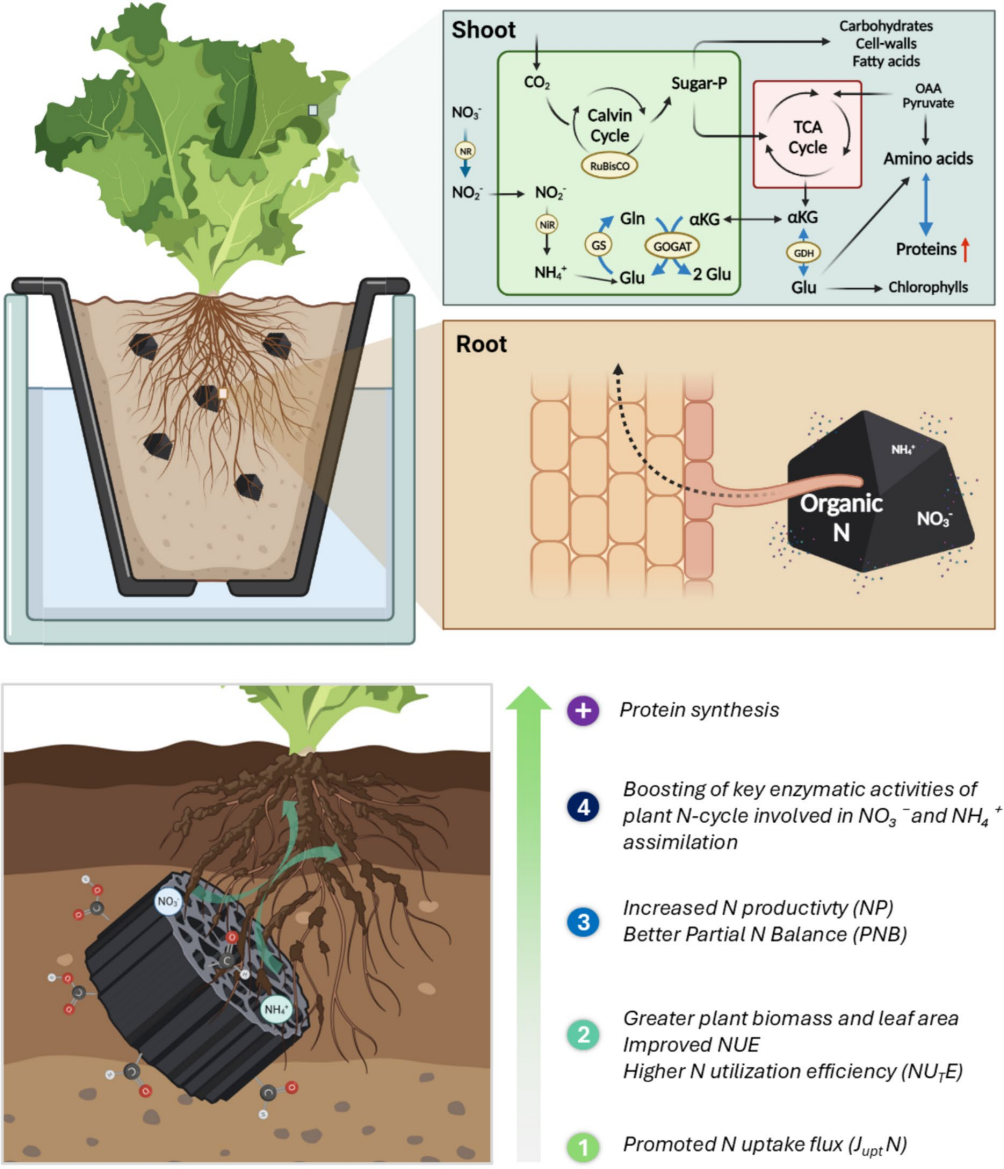
Proposed integrative model of N transport from roots and N assimilation in shoots of B30-grown plants, promoting protein synthesis. Created with Bio-render software. (Two-column image)

As shown in the model in Fig. 4, NR is the first and rate-limiting key enzyme in the NO₃⁻ assimilation, responsible for the reduction of NO₃^−^ to NO_2_^−^ in the cytosol (Fig. 3A). In our study, BC amendment significantly improved the NR activity in the leaves, suggesting higher NO₃⁻ assimilation. Since the accumulation of NO_2_^−^ is potentially toxic for the plant, it must be rapidly detoxified in the chloroplast by NiR into NH₄⁺ (Fig. 4). The NiR activity did not differ among treatments (Fig. 3B), which is consistent with its constitutive expression previously reported under biofortification experiments with nutrients in lettuce or tobacco plants (Blasco et al. 2010; Ríos et al. 2010; Peinado-Torrubia et al. 2023). However, other studies pinted to the view that NiR responses to BC may be dose-dependent and influenced by interactions with BC-N fertilization amounts (Khan et al. 2022). Thus, excessive BC can have the opposite effect, highlighting the importance of optimizing application rates for maximum benefit.

Consistent with the enhanced NR activity, downstream enzymes of the N assimilation pathway were also affected (Fig. 3). Following NO₃⁻ reduction, NH₄⁺ is assimilated by the GS/GOGAT cycle, which represents the key pathway for NH₄⁺ incorporation into organic compounds (Peinado-Torrubia et al. 2023). The GS catalyses the formation of glutamine (Gln) from NH₄⁺ and glutamate (Glu), and GOGAT subsequently transfers the amino group from Gln to 2-oxoglutarate (2-OG), producing two Glu molecules (Fig. 4; Harrison et al. 2000). While one Glu is used for the regeneration of the GS/GOGAT cycle, the other serves for the synthesis of amino acids, and other N-containing compounds. In line with the observed NR results, BC addition significantly increased both GS and GOGAT activities (Fig. 3C-D), indicating enhancing N assimilation capacity. Additionally, the activity of GDH, which also contributes to Glu biosynthesis by converting 2-OG, was higher in BC-treated plants (Fig. 3E). Since 2-OG derives from the TCA cycle, this pathway provides an alternative entry point for the incorporation of N into amino acids and peptides. These enzymatic changes reflect a coordinated upregulation of the N assimilation pathway in response to BC.

The combined upregulation of these N-related enzymes most likely contributed to the higher soluble protein content observed in B30 plants (Fig. 3F). This is further supported by the reduction in NH₄⁺ contents observed for the shoots, together with stable NO₃⁻ and organic N levels (Table 4), suggesting that inorganic N was rapidly converted into proteins rather than accumulated in its free forms. In addition, the decrease in NO₃⁻ levels in the roots, without a corresponding increase in the shoots, implies that NO₃⁻ was efficiently translocated and assimilated, avoiding foliar accumulation. The absence of differences in organic N content in the shoot may reflect a metabolic balance between N assimilation and its immediate incorporation into proteins and metabolic compounds. Although TN content (per g DW) decreased in roots or unchanged in shoots under BC treatments, the total N content per plant increased due to higher biomass accumulation (Fig. 1), indicating that increased NUE (Fig. 2B) and protein synthesis (Fig. 3F) resulted from both a more efficient metabolic conversion and indicative greater net N uptake. This interpretation is supported by the increase in TNA observed in B15 and B30 plants (Table 4), assessing a more effective incorporation of N into plant tissues.

Together, these findings suggest that BC enhances the efficiency of N use not only by increasing total N accumulation through greater biomass production, but also by promoting the enzymatic pathway involved in N assimilation. Previous studies reported that BC application may alter the internal distribution of N within the plant, favouring protein synthesis over the accumulation of free amino acids (Younis et al. 2015; Abd Elwahed et al. 2019; Huang et al. 2022). In our study, the increased protein content in the shoot was induced by the BC application, despite lower NH₄⁺ and total N, and unchanged NO₃⁻ and organic N levels, indicating a rapid and efficient metabolic incorporation of available inorganic N into organic compounds, particularly proteins. However, the underlying regulatory mechanisms that trigger this metabolic shift remain unclear and require further research.

### BC implications on the nitrogen assimilation in the substrate-root-shoot system

The impact of BC in N cycling and dynamics has been largely studied, and a substantial body of literature has accumulated over the last decades. Most of these studies explain the improved plant performance after BC addition by preventing N leaching and volatilization, due to the adsorption and retention of labile N–forms within the BC structure. Acting as a slow-release fertilizer, the immobilized N then can be gradually released and absorbed by the plant when needed. However, our study indicates that additional mechanisms can be involved beyond this well-established function.

One of those mechanisms may be related to the soil microbiome, which can modify the fertilizer status either by N immobilization through a facilitation of the sorption of N to the BC (Gou et al. 2023) or its immobilization into microbial biomass or by N mobilization through organic matter degradation processes. According to Ibrahim et al. (2020), BC-fertilizer interactions generally reduced bacterial diversity compared to BC alone, but this does not necessarily impair plant performance. They observed an increase of the proportion of Gammaproteobacteria, known as NO₃⁻ assimilator, by BC-fertilizers which may indicate that a good part of the NO₃⁻ can be converted back to NH₄⁺. This N-form can be immobilized or sorbed to BC surfaces and later remobilized for plant uptake.

In line with our findings, other studies have also revealed that BC is able to increase the activity of key N assimilation enzymes, as observed in rapeseed and cabbage (Huang et al. 2022; Khan et al. 2022). As discussed above, this enzymatic upregulation may lead to higher chlorophyll synthesis and photosynthesis, ultimately contributing to biomass gain. Thus, Peinado-Torrubia et al. (2023) showed that enhanced NR activity in tobacco correlates with increased chlorophyll content and photochemical efficiency, linking improved N assimilation with higher photosynthetic performance. This supports the idea that BC-driven upregulation of N metabolism may contribute to greater biomass through enhanced photosynthesis. Recently, Yang et al. (2024) demonstrated enzymes involved in starch and sucrose metabolism were upregulated under 600 kg ha^−1^ BC application, enhancing photosynthesis, C assimilation and therefore, and biomass accumulation in tobacco. However, excessive BC doses might disturb the source‒sink balance and hinder growth, underscoring the importance of optimizing BC application rates. Interestingly, Gao et al. (2023) identified four organic components in BC that could form H bonds with N-related transport proteins, ultimately leading to improved N assimilation and NUE in rice seedlings.

Only few studies have addressed the impact of BC on C and N metabolic routes. Farhangi-Abriz and Torabian (2018) found that BC improved N assimilation enzymes and RuBisCO activity in soybean under salt stress. RuBisCO, located in chloroplasts, drives the synthesis of sugar-phosphates, later incorporated into carbohydrates, cell walls and fatty acids (Zhao et al. 2024). Similarly, Tartaglia et al. (2020) reported higher abundance of energy and C metabolism proteins, as RuBisCO, in tomato after BC application, while stress-related proteins were less affected. This may result from enhanced the photosynthetic carboxylation in C_3_ plants compared to C_4_ plants when amended with BC (He et al. 2020). Huang et al. (2022) suggested that BC-based molybdenum directs more C toward amino acid synthesis, rather than other sugar-phosphate pathways, favoring protein formation. In our study, we can only state that, most N detected in shoots is organic N (i.e. amino acids, peptides, proteins). Thus, higher N content in plant biomass indicates greater assimilation of N for biomass creation and crop production.

## Conclusions

The findings of this study hold significant environmental and horticultural value, as they offer important insights and links for the interaction between BC and N dynamics in the substrate-root-shoot system. A major outcome of this study is that wood-derived BC, when incorporated up to 30% in a garden substrate, can considerably improve lettuce productivity and NUE parameters. Whereas previous studies related such effects mainly to N retention via sorption to BC surfaces, our results demonstrate that, in addition, BC stimulates the plant’s internal N-cycle by enhancing the activity of key enzymes involved in NO₃⁻ and NH₄⁺ assimilation. This metabolic enhancement contributes to more efficient use of available N and greater protein accumulation in shoot tissues, even under reduced total N concentrations. These findings offer promising perspectives for optimizing BC-based fertilizers, not just as passive N-retaining agents, but as enhancers of physiological N metabolism. However, whether this impact is caused directly by BC components, indirectly through changing physic and chemical soil/substrate conditions or mediated through plant–microbe interactions cannot be extracted from our study and certainly needs further research.

## Supplementary Information

Below is the link to the electronic supplementary material.

Supplementary file1 (DOCX 7485 KB)

## Data Availability

The datasets generated during this study are available from the corresponding author (M.A. Rosales), first author (A.F. Garcia-Rodriguez) or H. Knicker on reasonable request.

## Abbreviations

2-OG: 2-Oxoglutarate
BC: Biochar
GDH: Glutamate dehydrogenase
Gln: Glutamine
Glu: Glutamate
GOGAT: Glutamate synthase
GS: Glutamine synthase
J_upt_ N: N Uptake Flux
LA: Leaf Area
NiR: Nitrite reductase
NP: N Productivity
NR: Nitrate reductase
NUE: N Use Efficiency
NU_P_E: N Uptake Efficiency
NU_T_E: N Utilization Efficiency
PNB: Partial N Balance
RuBisCO: Ribulose-1,5-bisphosphate carboxylase/oxygenase
S: Succulence
SLA: Specific Leaf Area
TNA: Total N accumulation in the plant
TN_Plant_: Total N content detected in the plant
TN_Soil_: Total N detected in the soil or substrate
WC: Water content
