# Supplementary material for: Biochar enhances nitrogen use efficiency in lettuce by promoting its metabolic assimilation
Source: Plant Soil. 2025 Oct 31;518(1):299–317. doi: 10.1007/s11104-025-07997-0 (PMC12830439; doi:10.1007/s11104-025-07997-0)
Supplement: Supplementary file 1 — Supplementary file1 (DOCX 7485 KB) [file 11104_2025_7997_MOESM1_ESM.docx]

**Fig. S1** (A) Lettuce plants grown in peat:BC substrates at 25 DAS. (B0: 0% BC; B15: 15% BC, B30: 30% BC). (B) BC derived from green waste of vineyard prunings used in experimental treatments. Days after sowing (DAS); Biochar (BC).
